# Inverted Molding with Porous Skeleton Nickel Foam for Preparing Flexible Multi-Wall Carbon Nanotubes Pressure Sensors

**DOI:** 10.3390/s23239560

**Published:** 2023-12-01

**Authors:** Ruijie Liao, Xuhui Zhao, Mengran Liu

**Affiliations:** Hubei Key Laboratory of Modern Manufacturing Quantity Engineering, School of Mechanical Engineering, Hubei University of Technology, Wuhan 430068, China; 2210111132@hbut.edu.cn (R.L.); z15111939721@163.com (X.Z.)

**Keywords:** pressure sensor, MWCNTs, inverting molding, application

## Abstract

The application of traditional pressure sensors in health monitoring is limited by their initial rigidity. Flexible pressure sensors have thus received extensive attention owing to their excellent device flexibility. In this paper, we demonstrate a method of constructing flexible pressure sensors by inverting porous skeleton nickel foam based on multi-wall carbon nanotubes (MWCNTs) and polydimethylsiloxane (PDMS). MWCNTs and PDMS were mixed to form a composite conductive film, and the mass fraction of MWCNTs was optimized by evaluating the resistance change rate of the composite film. The optimized value of the mass fraction was 5%, which was used to prepare the flexible pressure sensors. The response, hysteresis, and stability of the sensors were further characterized. Pulse signals of humans were detected through flexible sensors, which can be used to evaluate cardiovascular conditions of the human body. These performance characteristics and the application demonstration show that our flexible pressure sensors have good prospects in human health care.

## 1. Introduction

In recent years, the rapid development of intelligent manufacturing, the industrial Internet of Things, intelligent medical applications, etc., has become inseparable from sensors. Sensors, computers, and communication are the three foundations of modern information technology. Among these, pressure sensors, which convert external pressure into electrical signals, have broad application prospects in daily life and industrial engineering [1,2]. In most complex scenarios, such as human-computer interaction and health monitoring, traditional pressure sensors are limited due to their initial rigidity. Flexible pressure sensors can be stretched, compressed, or folded, showing an ability to conformally maintain contact with curved surfaces and even human skin [3,4,5,6]. The working principles of these devices can be divided into resistance- [7], capacitance- [8,9], and voltage-based ones [10]. Various types of flexible pressure sensors with excellent electrical response characteristics and stability are in great demand in the fields of human health monitoring and human–computer interaction.

The main parts of flexible pressure sensors are the substrate and the pressure-sensitive film. Substrates generally used in flexible sensors include polymers, such as PDMS [11], polyimide (PI) [12], polyurethane (PU) [13], styrene-ethylene-butylene-styrene block copolymer (SEBS) [14], aliphatic-aromatic random co-polyester (Ecoflex) [15], etc. Among these, PDMS has been widely used, benefitting from its high elasticity, good durability, stable properties, good breathability, and biocompatibility [16]. 

For the pressure-sensitive film, Zhao et al. [17] prepared a porous carbon nanotube (CNT) sponge as a conductive film through a chemical vapor deposition (CVD) process. When a CNT sponge is compressed, the contact resistance of the porous film will change, achieving a response range of 100 KPa and an ultra-high sensitivity of 4015.8 KPa^−1^. However, the device response gradually deteriorated after 5000 test cycles, showing poor stability. Han et al. [18] electrostatically spun a mixture of polyacrylonitrile/dimethylformamide/AlCl_3_. It was then supported by PDMS after a process of pre-oxidization and carbonization. The device sensitivity remained stable after more than 25,000 cycles. However, its response range was small, with a maximum value of 5 Kpa, and the device sensitivity was poor, with a value of 1.41 KPa^−1^. Li et al. [19] prepared a TPU/ILs dielectric layer with a micropillar structure through an inverted molding method. Silver nanowires were deposited on semi-cured PDMS, producing a bilayer capacitive-type flexible pressure sensor. Under external pressure, the micropillars suffered from deformation, generating charges between the upper and lower surfaces of the dielectric layer. The capacitance change eventually occurred. The response range and sensitivity of the sensor were 170 KPa and 87.75 KPa^−1^, respectively. However, the sensitivity rapidly declined under medium and high pressures. Thus, flexible sensors with a wide response range and a high sensitivity in the whole range are significant. 

The main performance parameters of pressure sensors are sensitivity, response speed, response range, hysteresis, and stability [19,20]. Researchers have made breakthroughs in the device structure [21], the preparation processes [22], and the functional materials of the sensitive layers [23,24], improving the sensitivity of flexible pressure sensors to a great extent. However, most of the preparation processes are based on photolithography and plasma etching, which are complex and high-cost procedures. Moreover, accounting for the response range and sensitivity of the pressure sensor simultaneously was difficult. Preparing flexible pressure sensors with high sensitivity and stability through a simple and low-cost manufacturing process makes sense.

In this paper, PDMS and MWCNTs were adopted as the substrate and conductive material, respectively, and mixed together to form a composite conductive film. The porous skeleton structure of the nickel foam was used as a sacrificial template to prepare flexible MWCNT-PDMS pressure sensors. The mass fraction of MWCNTs was optimized by evaluating the resistance change rate of the composite film. The optimized value of the mass fraction was 5%, which was used to prepare the flexible pressure sensors. The response, hysteresis, and stability of the sensors were further characterized. Pulse signals of humans were detected through flexible sensors, which can be used to evaluate the cardiovascular conditions of the human body. These performance characteristics and the application demonstration show that our flexible pressure sensors have good prospects in human health care.

## 2. Preparation of Flexible Pressure Sensor

To prepare the porous composite films of the flexible pressure sensors, MWCNTs and PDMS were used as precursors and mixed. The MWCNTs and PDMS were provided by Jiangsu Xianfeng Nanomaterials Sci. & Tech. Co., Ltd. (Nanjing, China). and Dow Corning, Inc. (Midland, MI, USA), respectively. During the mixing, cyclohexane was used as a solvent to prevent MWCNTs from agglomerating.

**Figure 1:**
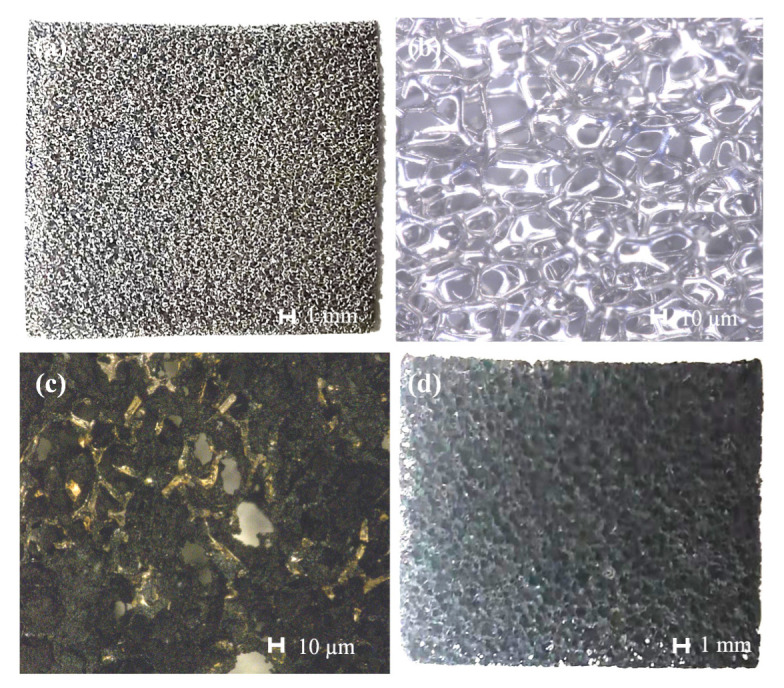
Nickel foam and MWCNT-PDMS porous film: (**a**) the optical image and (**b**) a magnified view of the nickel foam, (**c**) an optical image of the residual nickel foam, and (**d**) porous MWCNT-PDMS composite film.

A mixture of 5 g of PDMS and 10 mL of cyclohexane was stirred with a magnetic stirrer (KER-140GC, Wuhan Cole Instrument Co., Ltd., Wuhan, China) for 30 min to obtain a PDMS cyclohexane solution. A total of 0.25 g of MWCNTs was stirred in 10 mL of cyclohexane with a magnetic stirrer for 30 min and ultrasonically dispersed using an ultrasonic cell breaker (JY96-IIN, Ningbo Xinzhi Biotech Co., Ltd., Ningbo, China) for 30 min. To prevent the MWCNTs from agglomerating due to the thermal energy from the ultrasonication process, the solution was immersed in ice water. The well-dispersed MWCNTs were mixed with the PDMS cyclohexane solution and magnetically stirred for 1 h. To volatize the cyclohexane, the solution was magnetically stirred at room temperature for about 15 h. Next, a piece of porous nickel foam was introduced as the sacrificial template and cut into 10 × 10 mm^2^ sections. Figure 1a,b display an optical image and magnified view of the nickel foam, respectively. 

**Figure 2:**
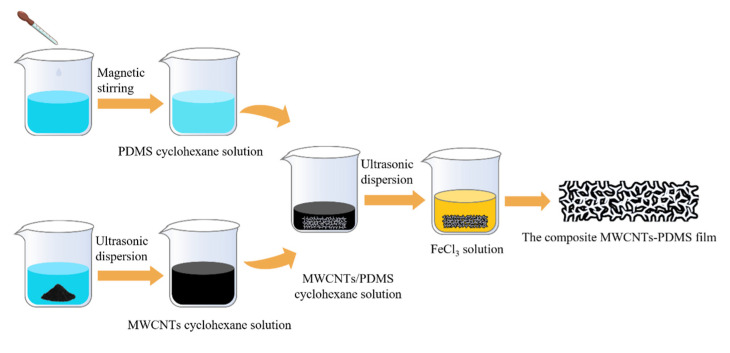
The whole process of preparing the MWCNT-PDMS films.

The FeCl_3_ solution was obtained by adding 27 g of ferric chloride hexahydrate (10025-77-1, Shanghai McLean Biochemical Technology Co., Ltd., Shanghai, China) to 89 mL of deionized water and magnetically stirring the mixture at 80 °C for 30 min. A piece of nickel foam was then immersed in the FeCl_3_ solution for 10 min to remove the surface-oxidized layer. The residual part of the solution was ultrasonically cleaned with deionized water using an ultrasonic cleaner (SB-5200DT, Kunshan Ultrasonic Instrument Co., Ltd., Kunshan, China) for 30 min. Next, the mixture of MWCNTs and PDMS was immersed in the dried nickel foam film and placed into an oven at 80 °C for 60 min to allow the PDMS prepolymer to completely solidify. Finally, the nickel foam containing the mixture of the MWCNTs and PDMS was immersed in the FeCl_3_ solution at 60 °C for about 10 h to fully dissolve the skeleton of the nickel foam. Figure 1c shows an optical image of the residual nickel foam skeleton. Figure 1d depicts the porous MWCNT-PDMS composite film attached to the skeleton of the nickel foam. Figure 2 demonstrates the whole process of preparing the composite MWCNT-PDMS films.

For the construction of the flexible pressure sensor, copper-nickel conductive fibers were used as the bottom and top electrodes. 

## 3. Results and Discussion

### 3.1. Characteristics of the MWCNT-PDMS Composite Films

#### 3.1.1. Conductivity of the MWCNT-PDMS Composite Films 

The conductivity of the MWCNT-PDMS composite films was mainly influenced by the mass fraction of the MWCNTs. A four-probe resistance tester was used to measure the square resistance *R_s_* of the films, based on which the conductivity *σ* could be calculated using the following equation:

(1) $$\sigma =\frac{1}{R_{S}t}$$

where *t* is the thickness of the composite films. The resistance of films with a size of 10 × 10 × 1 mm^3^ can be derived as follows:

(2) $$\sigma =\frac{l}{RS}$$

where *R* is the resistance of the composite film, *l* is the thickness of the film (1 mm), and *S* is the film area (100 mm^2^).

**Figure 3:**
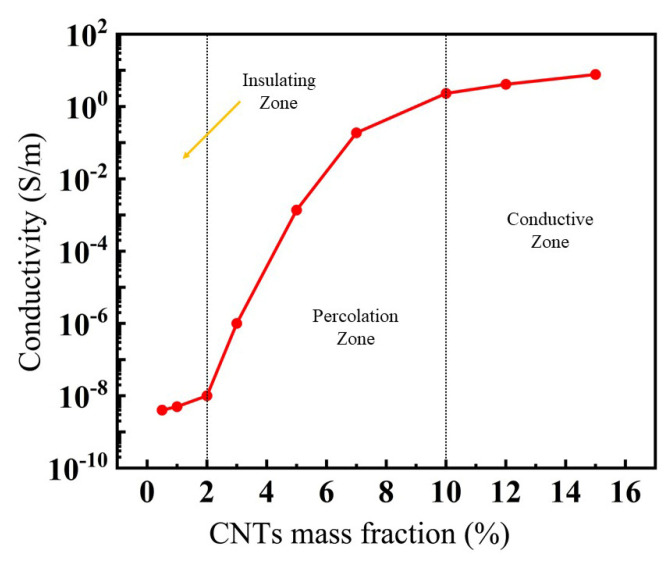
Variation trend of electrical conductivity vs. the CNT mass fraction.

Composite films with different mass fractions of MWCNTs were prepared first. Curves of conductivity vs. the mass fraction of MWCNTs were plotted, as shown in Figure 3. The curve was S-shaped. When the value of the mass fraction was less than 2%, conductive pathways did not form in this condition, thus leading to a conductivity of less than 10^−8^ S/m. When the mass fraction was more than 2%, the MWCNTs within the film came into direct contact with each other, thus making the conductivity increase rapidly when the mass fraction was enlarged. This region is called a percolation zone. When the mass fraction was larger than 10%, the film was in a conductive zone, where the conductivity was almost unaffected by the mass fraction, maintaining a value of 10 S/m. Moreover, when the mass fraction was below 5%, a small increase in the mass fraction resulted in a drastic enhancement in the film conductivity. Film deformation always occurs and causes significant changes in the conductivity. Thus, an obvious change in device currents will occur under a bias voltage. In this paper, porous MWCNT-PDMS composite films with a mass fraction of 5% were prepared and investigated.

#### 3.1.2. Microstructure of the MWCNT-PDMS Composite Film

**Figure 4:**
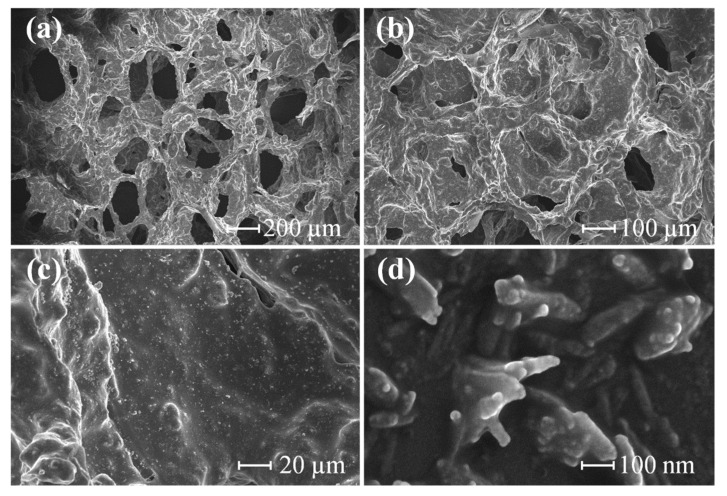
SEM images of composite films at different magnifications: (**a**) 50×, (**b**) 100×, (**c**) 500×, and (**d**) 80,000×.

Figure 4a,b show the SEM images of the porous MWCNT-PDMS composite films under magnifications of 50× and 100×, respectively. One can conclude that the MWCNT-PDMS precursor was wrapped around the nickel foam skeleton after the process. Figure 4c,d depict the scanning electron microscope (SEM) images of the composite films under magnifications of 500× and 80,000×, where the MWCNTs are distributed on the surface of the PDMS. Conductive pathways will be formed when the MWCNTs contact each other under external pressures.

### 3.2. Perfromace Characteristics of Flexible Pressure Sensors

#### 3.2.1. Response of the Flexible Pressure Sensors

**Figure 5:**
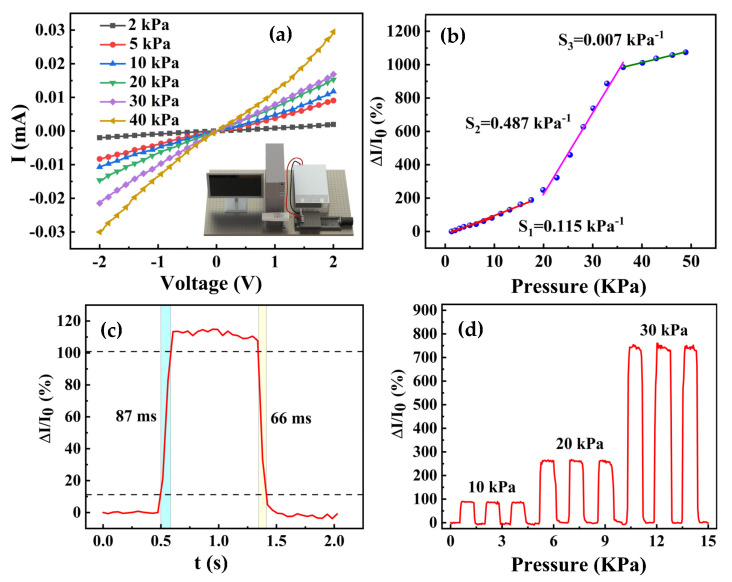
Flexible pressure sensors’ current variation test results, response curve, and hysteresis characteristics: (**a**) I–V curves under different pressures, (**b**) curves for change rate of the film resistivity, (**c**) a curve of response times, and (**d**) response curves under gradient pressures.

A system for the measurement of the pressure sensors was constructed, as illustrated in the inset of Figure 5a. During measurements, the sensor was fixed at the end of the uniaxial displacement platform. A digital source meter was connected to the electrodes of the sensor to collect the device resistances in real time. A push-pull gauge was fixed on the vibration-damping table to provide external pressures. Figure 5a displays the I–V curves of the sensor under different external pressures. One can conclude that the slope of the curves gradually increased when the pressure was enlarged, indicating that the state of the sensor changes rapidly after being pressed. The current change rates of the pressure sensor under different pressures were further concluded, as plotted in Figure 5b. The response sensitivity *S* of the pressure sensor can be calculated as follows:

(3) $$S=\frac{I-I_{0}}{I_{0}P}$$

where *I*_0_ and *I* are the current of the sensor in the initial state and under external pressure, and *P* is the value of the pressure. 

The relationship between the change rate of the film resistivity and the external pressures displayed a three-segment linear curve in the range of 0–50 kPa. In segment I (0–20 kPa), the resistivity change was minor due to a slight deformation, leading to a small sensor sensitivity S_1_ of 0.115 kPa^−1^. In segment II (20–35 kPa), the deformation of the composite film was obvious under increasing pressure, thus making the resistivity of the MWCNTs decrease. Conductive pathways can be formed by an electronic leap or direct connection of the MWCNTs, leading to a rapid change in the film conductivity with a sensor sensitivity S_2_ of 0.487 kPa^−1^. In segment III (35–50 kPa), the MWCNTs within the film formed a conductive network in the initial state without external pressures. The value of the film conductivity was almost unaffected under external pressures; thus, the change rate of the film resistivity was close to zero (0.007 kPa^−1^). 

The response time of the sensor was also characterized under a pressure of 10 kPa. The rise and fall times are shown in Figure 5c, with values of 87 and 66 ms, respectively, demonstrating our sensor has a rapid response. Detailed responses of the sensors under gradient pressures (10, 20, and 30 kPa) are shown in Figure 5d, proving the significant response repeatability of the sensor.

#### 3.2.2. Hysteresis and Stability of the Flexible Pressure Sensors

**Figure 6:**
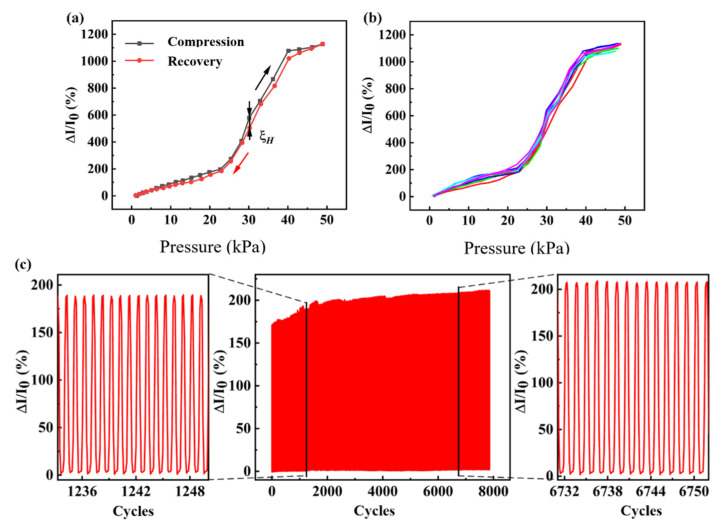
The hysteresis and stability of the flexible pressure sensors: (**a**) hysteresis curve, (**b**) hysteresis curve repeatability with the testes distinguished by the colors, and (**c**) stability curves.

The hysteresis of the sensor was characterized for external pressures from 0 to 50 KPa. The curves are shown in Figure 6a, where the value of the hysteresis ($\xi_{H}$) can be seen to be about 6.89%, indicating that the sensor has good hysteresis characteristics. As shown in Figure 6b, long-term measurements of the sensor under a periodic pressing and releasing process were also carried out. The hysteresis curves for five pressing and releasing processes are almost identical, indicating that conductive networks inside the sensor were not damaged during the processes, showing good consistency and repeatability.

The stability of the flexible pressure sensor under an external pressure of 20 KPa was tested through a uniaxial displacement platform. The current change rate of the sensor gradually held steady after a slight increase during the first several cycles. The beginning and ending cycles of the tests, as provided in Figure 6c, imply that the sensor maintains good stability during a long operation time.

The performance comparison between our work and previously reported research is provided in Table 1. From the results, one can see that our flexible pressure sensor obtained a good balance between the response range and sensitivity compared with the other devices based on CNTs/PDMS. The reason for this improvement may be the porous structure in the flexible pressure sensor introduced by the nickel foam. Under external pressure, film deformation can worsen under a porous structure, thus facilitating a high sensitivity. Moreover, a porous structure can also suffer from larger deformation than a flat film; that is why our flexible pressure sensor could obtain a wide response range.

### 3.3. Application and Experimental Testing of the Flexible Pressure Sensors

**Figure 7:**
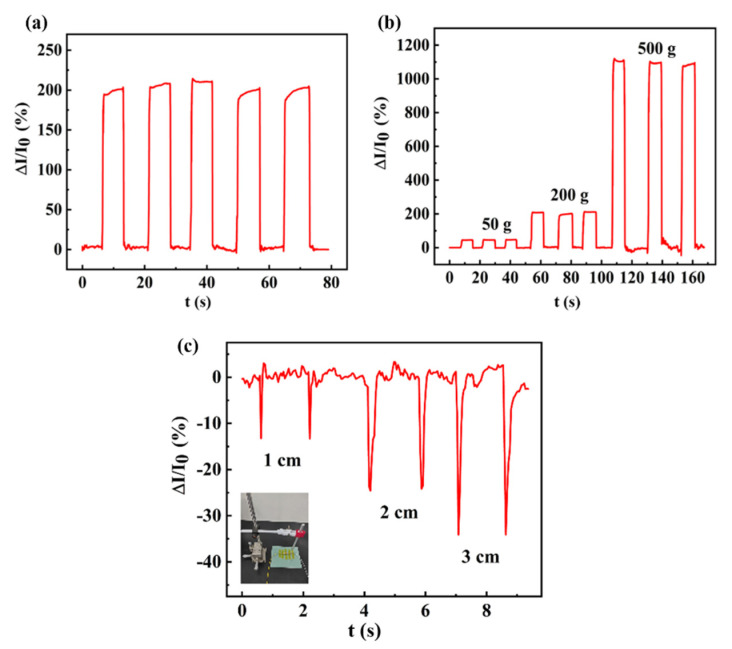
Application test of the pressure sensor: (**a**) response curve with 200 g weight, (**b**) response curve with 50 g, 200 g, and 500 g weights, and (**c**) vibration intensity detection with the inset depicting the simple drop test platform.

Applications of the pressure sensor were further explored. For example, periodic and obvious changes could be seen when a 200 g weight was applied and removed, as shown in Figure 7a. When 50, 200, and 500 g weights were introduced as external pressures on the sensor, the sensor demonstrated obvious current changes, as shown in Figure 7b. Further, flexible pressure sensors can also detect vibration signals generated by dropping objects. When the height from the objects to the surface of the sensor was adjusted from 1, 3 to 5 cm, the currents of the sensor significantly changed, as shown in Figure 7c. These results prove that the sensor may have good application prospects in the field of smart homes.

**Figure 8:**
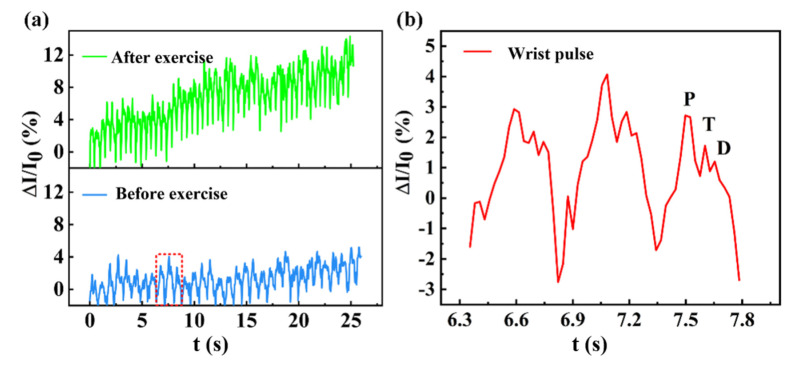
Pulse signal detected by the pressure sensor: (**a**) pulse signal before and after exercise and (**b**) magnified tiny pulse waveform for the detailed curves marked by the red square.

Pulse signals, as important physiological signals, play an important role in examining the state of the human cardiovascular system. The flexible pressure sensors in this manuscript were also demonstrated to detect tiny pulse signals. As shown in Figure 8a, the discrepancy in the pulse of humans before and after exercise can be clearly defined. From the results, it can be found that the frequency of the human pulse is about 74 beats/min in an initial state, and the value increases to 118 beats/min after exercise. The current change rate of the sensor was significantly enhanced after exercise. Figure 8b displays a magnified waveform of the pulse signals before exercise. Three peaks of the human pulse signal can be accurately detected, including the P-peak, T-peak, and D-peak. Based on these, the pulse wave conduction velocity, flexor artery reflective wave augmentation index, flexor artery diastolic augmentation index, and other physiological parameters can be derived to evaluate the cardiovascular condition of the human body. 

## 4. Summary

### 4.1. Parameter Results

The curves of the mass fraction vs. film conductivity were S-shaped. When the mass fraction of MWCNTs inside the composite film was less than 2%, a conductive pathway of MWCNTs could not easily form, and the film was in an insulating zone with a value below 10^−8^ S/m. When the mass fraction exceeded 2%, the MWCNTs in the film came into direct contact, forming a partially conductive MWCNT network. With a continuously increasing mass fraction, the conductivity of the composite film increased rapidly. Such a region is called a percolation zone. When the mass fraction reached 10%, an almost stable conductive network formed inside the film. The conductivity of the film remained stable with a value of 10 S/m with a further increase in the mass fraction. 

### 4.2. Measurement Results

During measurements, pressures were applied to the transducer by adjusting the displacement of the stage. The performance of the sensor was then characterized under gradient pressures (10, 20, and 30 kPa). The hysteresis of the sensor within 50 KPa was about 6.89%. The hysteresis curves of the sensor almost overlapped during five test processes.

### 4.3. Application Verifications

The flexible pressure sensors showed high responsivity and good stability. They can be also used to detect vibration signals when objects fall onto their surface. They also showed the ability to monitor the pulse signal of humans.

## 5. Conclusions

In this paper, MWCNTs and PDMS were mixed to form a composite conductive film. Then, an inverted molding process was performed for the composite conductive film using the porous skeleton structure of nickel foam to prepare flexible MWCNT-PDMS pressure sensors. The mass fraction of MWCNTs was optimized by evaluating the resistivity change rate of the film, obtaining an optimized value of 5%. In this condition, deformation inside the device under external pressure resulted in a significant conductivity change in the MWCNTs, which thus led to an obvious current change under a bias voltage. The response, hysteresis, and stability of the sensors were further characterized. Pulse signals of humans were detected through the flexible sensor, which can be used to evaluate the cardiovascular condition of the human body. These performance characteristics and the application demonstration show that our flexible pressure sensors have good prospects in health care. 

## Figures and Tables

**Table 1:** Performance comparison between our work and previously reported research.

Device Structure	Response Range (KPa)	Sensitivity (KPa^−1^)	Response Time	Ref.
CNTs/rayon	100	0.00547	\	[25]
CNTs/PI	3380	1.55	100/80	[26]
CNTs/PDMS	1200	0.02	8	[27]
CNTs/PDMS	4	4015.8	120 ms	[18]
CNTs/PDMS	0.5	1.41	\	[9]
CNTs/PDMS	35	0.487	87/66 ms	This work

## Data Availability

The data that support the findings of this study are available from the corresponding author upon reasonable request.
